# Ethanolic extract of *Streblus asper* leaves protects against glutamate-induced toxicity in HT22 hippocampal neuronal cells and extends lifespan of *Caenorhabditis elegans*

**DOI:** 10.1186/s12906-017-2050-3

**Published:** 2017-12-28

**Authors:** Anchalee Prasansuklab, Krai Meemon, Prasert Sobhon, Tewin Tencomnao

**Affiliations:** 1 Program in Clinical Biochemistry and Molecular Medicine, Department of Clinical Chemistry, Faculty of Allied Health Sciences, Chulalongkorn University, Bangkok, 10330 Thailand; 2 Department of Anatomy, Faculty of Science, Mahidol University, Bangkok, 10400 Thailand; 3 Faculty of Allied Health Sciences, Burapha University, Chonburi, 20131 Thailand; 4 Age-related Inflammation and Degeneration Research Unit, Department of Clinical Chemistry, Faculty of Allied Health Sciences, Chulalongkorn University, Bangkok, 10330 Thailand

**Keywords:** *Streblus Asper*, HT22 cell; Glutamate toxicity, Apoptosis, Neuroprotection, Oxidative stress, Nrf2 pathway, Lifespan, *Caenorhabditis elegans*

## Abstract

**Background:**

Although such local herb as *Streblus asper* (family Moraceae) has long been recognized for traditional folk medicines and important ingredient of traditional longevity formula, its anti-neurodegeneration or anti-aging activity is little known. This study aimed to investigate the neuroprotective effect of *S. asper* leaf extracts (SA-EE) against toxicity of glutamate-mediated oxidative stress, a crucial factor contributing to the neuronal loss in age-associated neurodegenerative diseases and the underlying mechanism as well as to evaluate its longevity effect.

**Methods:**

Using mouse hippocampal HT22 as a model for glutamate oxidative toxicity, we carried out MTT and LDH assays including Annexin V-FITC/propidium iodide staining to determine the SA-EE effect against glutamate-induced cell death. Antioxidant activities of SA-EE were evaluated using the radical scavenging and DCFH-DA assays. To elucidate the underlying mechanisms, SA-EE treated cells were analyzed for the expressions of mRNA and proteins interested by immunofluorescent staining, western blot analysis and quantitative real-time reverse transcription polymerase chain reaction (qRT-PCR) techniques. The longevity effect of SA-EE was examined on *C. elegans* by lifespan assay.

**Results:**

We demonstrate that a concentration-dependent reduction of glutamate-induced cytotoxicity was significant after SA-EE treatment as measured by MTT and LDH assays. Annexin V-FITC/propidium iodide and immunofluorescent staining showed that co-treatment of glutamate with SA-EE significantly reduced apoptotic-inducing factor (AIF)-dependent apoptotic cell death. DCFH-DA assay revealed that this extract was capable of dose dependently attenuating the ROS caused by glutamate. Western blot analysis and qRT-PCR showed that nuclear factor erythroid 2-related factor 2 (Nrf2) protein levels in the nucleus, as well as mRNA levels of antioxidant-related genes under Nrf2 regulation were significantly increased by SA-EE. Furthermore, this extract was capable of extending the lifespan of *C. elegans*.

**Conclusions:**

SA-EE possesses both longevity effects and neuroprotective activity against glutamate-induced cell death, supporting its therapeutic potential for the treatment of age-associated neurodegenerative diseases.

## Background

Neurodegeneration is an irreversible condition in which neurons gradually deteriorate and lose function, leading to impaired cognitive abilities, such as learning and memory. The most common and well-known type of neurodegenerative diseases is Alzheimer’s disease (AD) which accounts for approximately two thirds of all cases, and is associated with age. As aging populations increase rapidly, the number of patients continues to rise, in the absence of an effective treatment. The most commonly used AD drugs target acetylcholinesterase (AChE), but debate over their clinical efficacy and cost effectiveness has existed for decades, and they do not benefit all patients [1].

Efforts searching for new AD treatment has lately been focusing on reducing glutamate-induced oxidative toxicity. This mechanism has been implicated in many aging-associated disorders, particularly neurodegenerative diseases [2]. Disturbance of the glutamatergic system is one of the age-related physiological changes that could play an important role during neurodegeneration [3], as well as the normal process of aging [4, 5]. Glutamate is the principal excitatory neurotransmitter in the central nervous system (CNS). It is normally stored intracellularly and is known to be involved in a variety of normal brain functions including cognition. However, an increase in extracellular glutamate level can contribute to neuronal cell death via impaired mitochondrial function and the accumulation of reactive oxygen species (ROS) [6]. Hence, targeting towards glutamate-mediated toxicity pathways may offer another new approach for therapy of neurodegeneration. In addition, because of the serious side effects of current synthetic drugs, natural products from herbs or plant extracts could be a potential alternative intervention with good efficacy and minimal side effects for long-term treatment of this chronic disease.

*Streblus asper* Lour (SA) is a medicinal plant belonging to the family Moraceae and is mainly distributed in Asian countries. The traditional uses of almost every part of this plant are well documented in the Ayurveda and other traditional folk medicines for various medicinal purposes such as treatment of filariasis, leprosy, syphilis, fever, dysentery, diarrhea, piles, toothache, epilepsy, epistaxis, heart disease, ulcers, obesity, wounds, inflammatory swellings, and cancer [7, 8]. SA has been also been used as an ingredient in one popular Thai traditional formula for longevity [9]. So far, no or few scientific reports have confirmed the properties and mechanisms of this plant for anti-aging or anti-neurodegeneration [10]. However the plant leaves appear to have strong natural antioxidant properties [11, 12] that are capable of counteracting oxidative damage, and exerting protective effect in neuronal cells [10]. Moreover, some compounds previously characterized from SA such as flavonoids and lignans [13] have been shown to cross the blood-brain barrier [14–17]. Therefore, in this present study we set out, for the first time, to investigate the neuroprotective effect of an ethanolic leaf extract of SA (SA-EE) against glutamate toxicity, and also determined the underlying mechanism of neuroprotection in cultured mouse clonal hippocampal (HT-22) cells. Besides, its anti-aging activity was evaluated in vivo using the nematode *Caenorhabditis elegans* (*C. elegans*) as a model.

## Methods

### Chemicals and antibodies

L-glutamic acid, 2,2′-Azino-bis(3-ethylbenzothiazoline-6-sulfonic acid) diammonium salt (ABTS), 2,2-Diphenyl-1-picrylhydrazyl (DPPH), quercetin, curcumin, dimethyl sulfoxide (DMSO), Dulbecco’s modified Eagle’s medium (DMEM), fetal bovine serum (FBS), Quercetin and Folin-Ciocalteu phenol reagent were purchased from Sigma-Aldrich (St. Louis, MO, USA). 3-(4,5-dimetylthiazol-2-yl)-2,5-diphenyltetrazoliumbromide (MTT) was purchased from Bio Basic (Markham, Ontario, Canada). Hydrogen peroxide (H_2_O_2_; 30%) was purchased from Merck (Darmstadt, Germany). Gallic acid was purchased from TCI America (Portland, OR, USA). L-ascorbic acid was purchased from Calbiochem (San Diego, CA, USA). 2′, 7′-dichlorodihydrofluorescein diacetate (H_2_DCFDA) was purchased from Molecular Probes (Eugene, OR, USA). Trizol was purchased from Invitrogen (Carlsbad, CA, USA). Penicillin/Streptomycin solution was purchased from Gibco (Waltham, MA, USA). Antibodies against apoptotic-inducing factor (AIF), β-actin, Lamin B1, and secondary antibodies were purchased from Cell Signaling Technology (Danvers, MA, USA). The excitatory amino acid transporter 3 (EAAT3/EAAC1) and nuclear factor erythroid 2-related factor 2 (Nrf2) antibodies were purchased from Abcam (Cambridge, UK) and Santa Cruz Biotechnology (Dallas, Texas, USA), respectively. The CytoTox 96® kit for LDH assay was purchased from Promega (Madison, WI, USA). The annexin V-FITC apoptosis detection kit was purchased from BioLegend (San Diego, CA, USA). The NE-PER nuclear and cytoplasmic extraction reagents was purchased from Thermo Scientific (Rockford, IL, USA). RT PreMix and qPCR Master Mix solution were purchased from Bioneer (Daejeon, South Korea).

### Plant material and extract preparation

*S. asper* was collected from the Princess Maha Chakri Sirindhorn Herbal Garden (Rayong Province, Thailand). The plant was identified and its voucher specimen [A013419 (BCU)] was deposited in the herbarium of Kasin Suvatabhandhu (Department of Botany, Faculty of Science, Chulalongkorn University, Thailand). The leaves of *S. asper* were dried under shade and ground into fine powder. Successive extraction was carried out by soaking 35 g of dried powdered sample in absolute ethanol for 48 h at room temperature (RT). The sample was re-extracted twice and all resulting supernatants were combined, subsequently filtered and evaporated. Finally, about 1.41 g of the ethanol crude extract (SA-EE) was obtained. The extract was dissolved in DMSO as stock solution (100 mg/mL), passed through a 0.2-μm filter and stored at −20 °C until use.

### Cell culture

Mouse hippocampal HT22 cells (a generous gift from Professor David Schubert at the Salk Institute, San Diego, CA, USA) were maintained in DMEM supplemented with 10% (*v*/v) fetal bovine serum and 1% penicillin/streptomycin at 37 °C in a humidified incubator with 5% CO_2_. The cells were plated and incubated overnight prior to treatment with glutamate alone or in combination with different concentrations of SA-EE for the indicated times.

### Determination of cell viability

#### MTT assay

Cell viability was assessed by measuring MTT reduction capacity of mitochondrial enzymes in viable cells. The MTT solution was added to culture medium at a final concentration of 0.5 mg/mL and left in the dark for 4 h at 37 °C. Afterwards, all solution was removed and the formazan crystals were solubilized by DMSO-ethanol mixture (1:1, *v*/v). Absorbance was read at 550 nm using an EnSpire® Multimode Plate Reader (Perkin-Elmer, Waltham, MA, USA). Results are expressed as a percentage relative to untreated control.

#### LDH assay

Cell viability was evaluated by measuring lactate dehydrogenase (LDH) leakage from damaged cells due to the loss of cell membrane integrity. The activity of LDH release in culture medium was measured using the CytoTox 96® assay (Promega) according to manufacturer’s instructions. Briefly, the culture supernatant was incubated with substrate mix for 30 min in the dark at RT, followed by addition of stop solution. Absorbance was then recorded at 490 nm using an EnSpire® Multimode Plate Reader (Perkin-Elmer). Results are expressed as a percentage of maximum LDH release obtained by complete cell lysis.

### Flow cytometry with Annexin V/PI staining

Apoptotic cell death was quantified by flow cytometry using the FITC annexin V apoptosis detection kit with propidium iodide (PI) (BioLegend) according to manufacturer’s protocol. Briefly, the cells were harvested at the end of treatment, washed by phosphate-buffered saline (PBS), and re-suspend in binding buffer before staining with FITC-conjugated annexin V and PI solution for 15 min in the dark. Fluorescence intensity of stained cells was immediately analyzed using a BD FACSCalibur™ flow cytometer (BD Bioscience, Heidelberg, Germany). 1 mM H_2_O_2_-treated cells were used as the positive control [18]. Data were collected from at least 10,000 cells per group and results are expressed as the percentage of apoptotic cells.

### Immunofluorescent staining

The cells were fixed with cold 4% (*w*/*v*) paraformaldehyde in PBS for 20 min, rehydrated in PBS for 15 min, permeabilized in 0.1% (w/v) Triton X-100 in PBS for 10 min at RT, and blocked with 5% bovine serum albumin (BSA) in PBS for 30 min at RT. After being washed with PBS, the cells were incubated overnight at 4 °C with primary antibodies against AIF (1:400), followed by incubation with Alexa Fluor 555-conjugated goat anti-rabbit (1:2000) for 1 h at RT. The nuclei were counterstained with 4′,6-diamidino-2-phenylindole (DAPI) (300 nM). The images were captured using an LSM 700 confocal laser scanning microscope (Carl Zeiss, Jena, Germany).

### Western blot analysis

Whole cell lysates were prepared in NP-40 lysis buffer (50 mM Tris pH 8.0, 150 mM NaCl, 1% NP-40, 1 mM PMSF, 1 mM DTT). Cytoplasmic and nuclear fractions were isolated using the NE-PER nuclear and cytoplasmic extraction reagents (Thermo Scientific) according to the manufacture’s protocol. Total protein concentrations were quantified by the Bradford assay. Equal amount of protein (10 μg) were separated on 10% SDS-polyacrylamide gel and then transferred to PVDF membranes. After blocking for 1 h with 5% skim milk in TBS-T (Tris-buffered saline, 0.1% Tween 20), the membranes were allowed to incubate overnight at 4 °C with primary antibodies specific for Nrf2 (1:2000), EAAT3 (1:8000), AIF (1: 2000), Lamin B1 (1: 2000) or β-actin (1: 16,000), and subsequently with HRP-conjugated secondary antibodies (1:10,000) at room temperature for 45 min. Specific protein bands were visualized with enhanced chemiluminescence using the ECL Select western blotting detection reagent (GE Healthcare, Marlborough, MA, USA). Densitometric analysis of the bands was performed with the Syngene image analysis system (Cambridge, UK).

### In vitro evaluation of antioxidant properties

#### Radical scavenging activity assay

Free radical scavenging activity was measured using stable radical DPPH (DPPH•) and stable cation radical ABTS (ABTS•+). A solution of DPPH• was diluted in ethanol at a concentration of 0.2 mg/mL. The ABTS• + solution was prepared freshly on dilution with ethanol until the absorbance reached 0.7 to 0.8 at 734 nm. The reaction consisted of DPPH• or ABTS• + solution and the extract (1 mg/mL) diluted in the same solvent at a 9:1 ratio. After an incubation period of 15 min for DPPH assay or 30 min for ABTS assay at RT, the absorbance was read at 517 nm or 734 nm, respectively, using an EnSpire® Multimode Plate Reader (Perkin-Elmer). Ascorbic acid (vitamin C) at various concentrations was used as standard for both assays. Radical scavenging activity was expressed as the percent inhibition of the radical calculated by the following equation: %Inhibition = 100 - [(Abs of sample- Abs of blank) × 100/ Abs of control]. The antioxidant capacity was expressed as vitamin C equivalent antioxidant capacity (VCEAC) in mg per g of dry weight plant extract.

#### Assay for total phenolic content

The total phenolic content was determined by the Folin-Ciocalteu method modified for a microplate format. Briefly, 50 μL of the extract (1 mg/mL) was mixed thoroughly with 50 μL of a 10-fold diluted Folin-Ciocalteu’s phenol reagent. After 20 min, the mixture was neutralized by addition of 50 μL of a 7.5% (*w*/*v*) Na_2_CO_3_ solution and then kept in the dark at RT for a further 20 min. Finally, the absorbance was measured at 760 nm using an EnSpire® Multimode Plate Reader (Perkin-Elmer). Gallic acid was used as a standard for the calibration curve and total phenolic content was expressed as mg of gallic acid equivalent (GAE) per g of dry weight plant extract.

#### Assay for total flavonoid content

The total flavonoid content was determined by an aluminum chloride colorimetric method modified for a microplate format. In brief, 50 uL of the extract (1 mg/mL) was made up to 200 μL with 95% ethanol, and mixed well with 10 μL of 10% (*v*/v) AlCl_3_ solution and 10 μL of 1 M NaOAc solution. Then the mixture was allowed to stand for 40 min in the dark and the absorbance was measured at 415 nm. The total flavonoid content was calculated from a calibration curve using quercetin as a standard, and results are expressed as mg of quercetin equivalent (QE) per g of dry weight plant extract.

#### Intracellular ROS measurement

ROS production was quantified by the DCFH-DA method. The cell-permeant H_2_DCFDA fluorescent probe is commonly used to detect cellular production of ROS. After treatment, the cells were loaded with 5 μM H_2_DCFDA for 30 min at 37 °C, followed by washing three times with Hank’s balanced salt solution (HBSS). The fluorescence intensity (excitation = 485 nm; emission = 535 nm) was measured using an EnSpire® Multimode Plate Reader (Perkin-Elmer) and the photographs were obtained using an Axio Observer A1 fluorescence microscope (Carl Zeiss, Jena, Germany). Serving as the positive control, cells were treated with a well-known stress inducer, H_2_O_2_ at concentration of 1 mM [18]. Data are expressed as the percentage of fluorescence intensity of treated cells relative to untreated control.

### RNA isolation and quantitative RT-PCR

Total RNA was extracted using Trizol reagent (Invitrogen) following the manufacturer’s instructions. The amount of RNA was determined by absorbance at 260 nm. 1 μg of total RNA was used for cDNA synthesis using AccuPower RT PreMix (Bioneer) and oligo(dT)17 primer. All real-time PCR reactions were performed in an Exicycler™ 96 (Bioneer). The amplifications were done using the GreenStar™ qPCR PreMix (Bioneer) and specific primers for NQO1 (Forward: 5’-CGACAACGGTCCTTTCCAGA-3′, Reverse: 5’-CTCCCAGACGGTTTCCAGAC-3′), GCLM (Forward: 5’-GGAGCTTCGGGACTGTATCC-3′, Reverse: 5’-CAACTCCAAGGACGGAGCAT-3′), EAAT3 (Forward: 5’-ATGATCTCGTCCAGTTCGGC-3′, Reverse: 5’-TGACGATCTGCCC AATGCTT-3′), and β-actin (Forward: 5’-GGCTGTATTCCCCTCCATCG-3′, Reverse: 5’-CCAGTTGGTAACAATGCCATGT-3′) as a normalization control. The thermal cycling conditions were composed of an initial denaturation step at 95 °C for 10 min, followed by 40 cycles at 95 °C for 15 s, 55 °C for 15 s and 72 °C for 30s. A melting curve analysis was performed after amplification to verify the accuracy of the amplicon. Expression data was normalized to β-actin and expression levels were analyzed using the 2^-ΔΔCT^ method.

### Nematode strain, culture condition and lifespan assay

Wild-type Bristol N2 *C. elegans* strain was maintained at 20 °C on nematode growth medium (NGM) agar and fed with *Escherichia coli* OP50 as a food source. Before the experiment, an age-synchronized population at L1 larvae was obtained by treating gravid *C. elegans* hermaphrodites with sodium hypochlorite treatment to collect the eggs and growing them on NGM agar without bacteria at 20 °C overnight. To acquire L4 larvae, synchronized L1 larvae were transferred onto NGM plates containing *E. coli* OP50 and incubated for 40 h at 20 °C. For the lifespan assay, wild-type synchronized L1 or L4 larvae were plated on NGM agar plates containing a lawn of *E. coli* OP50 supplemented with different concentrations of the extracts. The worms were grown at 25 °C, counted daily, and scored as dead when they did not respond to gentle stimulus with a platinum wire and showed no pharyngeal pumping movement. Worms with internally hatched progeny or extruded gonads were censored and must exclude from the experiment. The experiment was performed with at least 100 worms per group.

### Qualitative phytochemical screening

The extracts were submitted to Institute of Systems Biology (Universiti Kebangsaan Malaysia, Malaysia) for screening of phytochemical constituents using Liquid Chromatography-Mass Spectrometry (LC-MS) analysis. The chromatographic separation was carried out on a Dionex™ UltiMate 3000 UHPLC system (Thermo Scientific) equipped with an Acclaim™ Polar Advantage II C18 column (3 × 150 mm, 3 μm particle size) (Thermo Scientific). Injection volume was 1 μL. The mobile phases were 0.1% formic acid in water (solvent A) and 100% acetonitrile (solvent B), which were allowed to pass through the column at a flow rate of 400 μL/min within 22 min total run time. The gradient elution started at 5% B (0-3 min); 80% B (3-10 min); 80% B (10-15 min) and 5% B (15-22 min). High resolution MS analysis was performed in the positive electrospray ionization mode using a MicrOTOF-Q III (Bruker Daltonik GmbH, Bremen, Germany). The instrument parameters were set as follows: nebulizer pressure at 1.2 bar, capillary voltage at 4500 V, drying gas flow at 8 L/min with the ion source temperature at 200 °C, end plate offset of −500 V, and scan range from m/z 50 to 1000. Putative compounds were identified by comparing the obtained m/z values with the METLIN and the KNApSAcK databases as well as with the calculated mass values from previously published data available, with a difference of less than 30 parts-per-million (ppm) was acceptable. Relative amount is determined as the percentage of peak area relative to total area of all peaks observed in the chromatogram.

### Statistical analysis

All experiments were performed in at least triplicate. The data are shown as the mean ± SEM. Comparison between two groups was analyzed by a two-tailed unpaired Student’s t-test. The differences of more than two groups were compared using one-way ANOVA analysis following the post hoc Tukey HSD test. For the lifespan assay, comparison of the survival distributions among different groups was done by a log-rank (Mantel − Cox) test. All statistical analyses were conducted using SPSS or GraphPad Prism software, with *P* < 0.05 considered to be statistically significant.

## Results

### SA-EE protects against glutamate-induced cytotoxicity

**Fig. 1:**
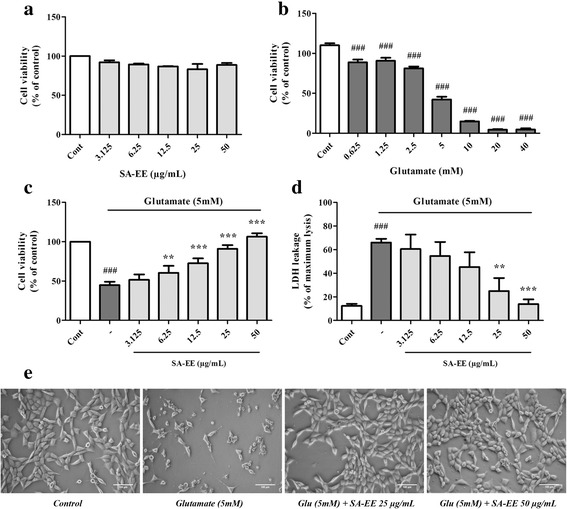
Protective effect of SA-EE against glutamate-induced toxicity in HT22 cells. Toxicity of SA-EE (**a**) and glutamate (**b**) in HT22 cells was determined by MTT assay after treatment at different concentrations for 24 h. Cells were exposed to 5 mM glutamate alone or glutamate in combination with different concentrations of SA-EE for 24 h and then cell viability was measured by MTT (**c**) and LDH (**d**) assay. Cell morphology was observed under microscope at 5X magnification (**e**). All data are shown as the mean ± SEM of at least three independent experiments. ^*###*^ *P* < 0.001 vs. control; **P < 0.01, ***P < 0.001 vs. glutamate alone

Exposure to varied concentrations of SA-EE did not cause noticeable toxicity to cells whose cell viability was above 80% (Fig. 1a). Treatment with different glutamate concentrations ranging from 0.625 to 40 mM induced neuronal cell death in a dose-dependent manner (Fig. 1b). Thus, the concentration of 5 mM resulting in a reduction of approximately 50% of the cells was chosen for subsequent experiments. In the presence of SA-EE, this glutamate-induced cell toxicity was significantly concentration-dependent reduced, as determined by MTT (Fig. 1c) and LDH (Fig. 1d) assays, as well as morphological examination (Fig. 1e). We found that 50 μg/mL of SA-EE was able to improve cell viability and restore LDH leakage to the control level. These results suggest that SA-EE exerts a potent neuroprotective effect against cytotoxicity induced by glutamate in cultured hippocampal neuronal cells.

### SA-EE protects against glutamate-induced oxidative stress

**Fig. 2:**
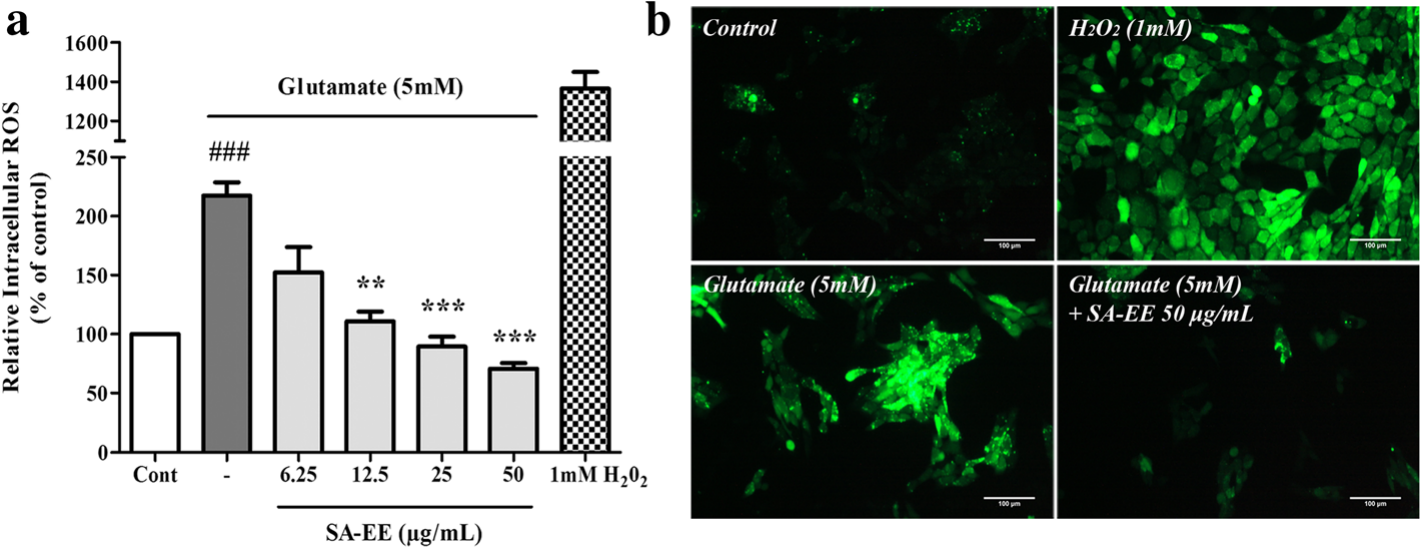
Protective effect of SA-EE against glutamate-induced oxidative stress in HT22 cells. HT22 cells were exposed to 5 mM glutamate alone or glutamate in combination with different concentrations of SA-EE for 14 h before staining with DCFH-DA probe. H_2_O_2_-treated cells were used as a positive control. Intracellular ROS production was measured using a fluorescent plate reader (**a**). Representative fluorescence micrographs of the cells stained with DCFH-DA was observed under a fluorescence microscope (**b**). All data are shownas the mean ± SEM of at least three independent experiments. ^*###*^ *P* < 0.001 vs. control; **P < 0.01, ***P < 0.001 vs. glutamate alone

Enhanced oxidative stress has been proposed as a major mechanism underlying the cytotoxic action of glutamate at high concentration that results in neuronal cell death. To investigate whether SA-EE could suppress glutamate-induced oxidative stress, we evaluated the antioxidant properties of SA-EE in vitro and in cells. Table 1 presents DPPH and ABTS radical scavenging activity, total phenolic and flavonoid contents of SA-EE. Intracellular ROS level measured by DCFH-DA assay showed approximately two-fold increase in cells exposed to 5 mM glutamate compared to control. However, co-treatment of SA-EE significantly decreased ROS generation caused by glutamate in a dose-dependent manner, with SA-EE at a concentration 50 μg/mL restoring ROS levels to that of control (Fig. 2). This result suggests that SA-EE protects against glutamate-induced cytotoxicity by suppressing intracellular ROS production.

**Table 1:** Total phenolic content, total flavonoid content and free radical scavenging capacity of SA-EE

Total Phenolics mg GAE/g dry weight sample	Total Flavonoids mg QE/g dry weight sample	DPPH scavenging assay		ABTS scavenging assay	
		%Radical Scavenging activity (of 1 mg/mL extract)	mg VCEAC/g dry weight sample	%Radical Scavenging activity (of 1 mg/mL extract)	mg VCEAC/g dry weight sample
26.89 ± 0.96	5.17 ± 1.17	15.37 ± 0.7	17.7 ± 0.39	56.5 ± 6.28	31.03 ± 3.98

Values are expressed as mean ± SD (*n* = 3)

### SA-EE promotes the cellular antioxidant defense via the Nrf2-dependent response

**Fig. 3:**
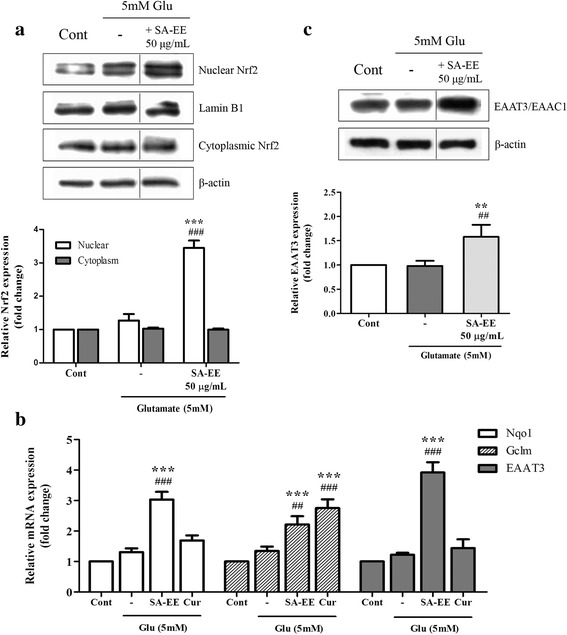
Effect of SA-EE on Nrf2 pathway activation. HT22 cells were exposed to 5 mM glutamate alone or glutamate in combination with 50 μg/mL SA-EE for 1 h prior to western blot analysis of nuclear and cytoplasmic Nrf2 levels (**a**). Quantitative real-time RT-PCR analysis of NQO1, GCLM and EAAT3 mRNA expression was performed after treatment of cells with 5 mM glutamate alone or glutamate in combination with 50 μg/mL SA-EE or 15 μM curcumin (positive control) for 24 h (**b**). Whole cell lysates were subjected to western blot analysis of EAAT3 level after treating cells with 5 mM glutamate alone or glutamate in combination with 50 μg/mL SA-EE for 24 h (**c**). Lamin B1 and β-actin served as endogenous loading controls for nuclear extracts and whole cell/cytoplasmic extracts, respectively. β-actin was used as an internal control for RT-PCR assay. All data were normalized to endogenous control levels and are expressed as the mean ± SEM of at least three independent experiments. ^*##*^ *P* < 0.01, ^*###*^ *P* < 0.001 vs. control; **P < 0.01, ***P < 0.001 vs. glutamate alone

To further elucidate the SA-EE’s mechanism of action in antioxidant-mediated neuroprotection against glutamate toxicity, we examined the effect of SA-EE on the Nrf2 signaling pathway using western blot and real-time RT-PCR analysis. Treatment of 50 μg/mL SA-EE with 5 mM glutamate caused rapid nuclear accumulation of Nrf2 without altering its cytoplasmic level (Fig. 3a). The level of Nrf2 expression in the nucleus significantly increased at 1 h after co-treatment of SA-EE and glutamate to 3.4- and 2.7-fold of the control and glutamate alone groups, respectively. In addition, SA-EE also induced the expression of several antioxidant-related target genes including NAD(P)H:quinone oxidoreductase 1 (NQO1), glutamate cysteine ligase complex modifier subunit (GCLM), and EAAT3 which are under Nrf2 regulation. Exposure to SA-EE or curcumin resulted in a significant increase in transcriptional expression of NQO1 and GCLM in glutamate-treated cells (Fig. 3b). Both protein and mRNA levels of EAAT3 were also significantly up-regulated by co-treatment with SA-EE when compared to control and glutamate alone (Fig. 3b and c). However, the expression of EAAT3 mRNA did not change with curcumin treatment. Collectively, these findings demonstrate that SA-EE promotes antioxidant defense by activating Nrf2 and its downstream regulated genes, providing a plausible mechanism for its neuroprotective effect.

### SA-EE protects against glutamate-induced cell apoptosis via caspase-independent pathway

**Fig. 4:**
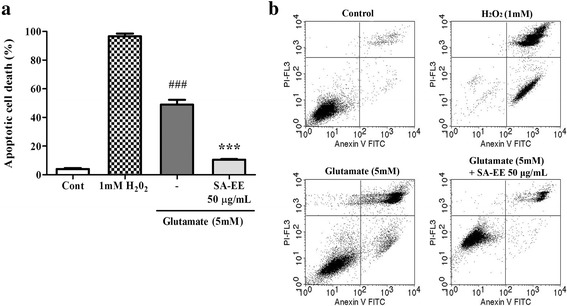
Quantitative flow cytometric analysis of apoptotic cells in HT22 cells. HT22 cells were exposed to 5 mM glutamate alone or glutamate in combination with 50 μg/mL SA-EE for 18 h before staining with FITC-conjugated annexin V and PI. H_2_O_2_-treated cells were used as a positive control. Percentages of apoptotic cells were detected using flow cytometric analysis of annexin V-positive/PI–negative cells (early stage, lower right quadrant) plus annexin V/PI–positive cells (late stage, upper right quadrant) (**a**). Representative scatter plots show the distribution of annexin V and PI staining for control and treated cells (**b**). All data are shown as the mean ± SEM of at least three independent experiments. ^*###*^ *P* < 0.001 vs. control; ***P < 0.001 vs. glutamate alone

**Fig. 5:**
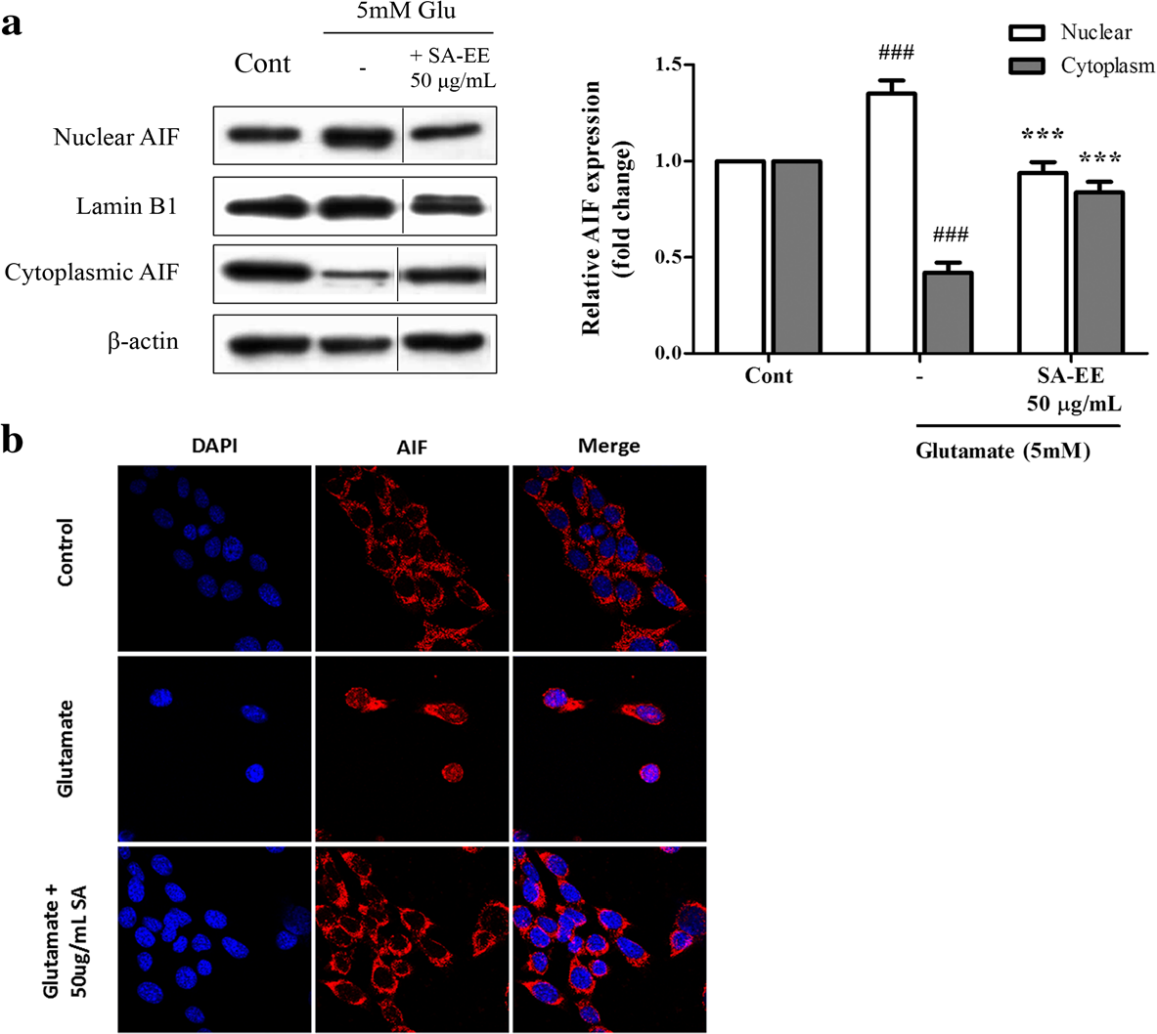
Protective effect of SA-EE against glutamate-induced AIF nuclear translocation in HT22 cells. HT22 cells were exposed to 5 mM glutamate alone or glutamate in combination with 50 μg/mL SA-EE for 16 h prior to western blot analysis of nuclear and cytoplasmic AIF levels (**a**). Lamin B1 and β-actin served as endogenous loading controls for nuclear extracts and whole cell/cytoplasmic extracts, respectively. All data were normalized to endogenous control levels and are expressed as the mean ± SEM of at least three independent experiments. ^*###*^ *P* < 0.001 vs. control; ***P < 0.001 vs. glutamate alone. Representative images of the cells stained with antibody against AIF (red) were observed under confocal microscope during treatment. The cells were counterstained with DAPI (blue) to indicate nuclear location (**b**)

Nuclear translocation of AIF is one of the major downstream mechanisms underlying glutamate-induced neuronal cell death mediated through ROS formation. Elevated intracellular ROS levels can induce release of mitochondrial AIF to nucleus, thereby triggering apoptosis in a caspase-independent manner [6]. Results of flow cytometric analysis using Annexin V/PI staining confirmed apoptosis inductionby glutamate treatment, with the majority of apoptotic cells in a late stage of apoptosis (Fig. 4b). However, approximately 40% of cells killed after exposure to 5 mM glutamate could be markedly rescued by co-treatment with 50 μg/mL of SA-EE (Fig. 4a). To further explore the protective mechanisms of SA-EE against glutamate-induced apoptotic cell death, we measured the subcellular distribution of AIF by immunofluorescence and western blotting. Following treatment with 5 mM glutamate, the AIF proteins were found significantly increased in the nucleus but decreased in the cytoplasm (Fig. 5a). Confocal microscopy taken at 16 h after glutamate exposure also revealed that AIF distributed in the cytosol under control condition was translocated into the nucleus after glutamate treatment (Fig. 5b). However, SA-EE at 50 μg/mL could prevent the accumulation of AIF in the nucleus and retained the cytosolic distribution (Fig. 5b). The nuclear AIF levels in the cells co-treated with SA-EE and glutamate were significantly reduced to an extent comparable to that observed in control cells (Fig. 5a). Taken together, these results indicate that the neuroprotective effect of SA-EE was through inhibition of a caspase-independent mechanism of apoptosis.

### SA-EE extends the lifespan of *C. elegans*

**Fig. 6:**
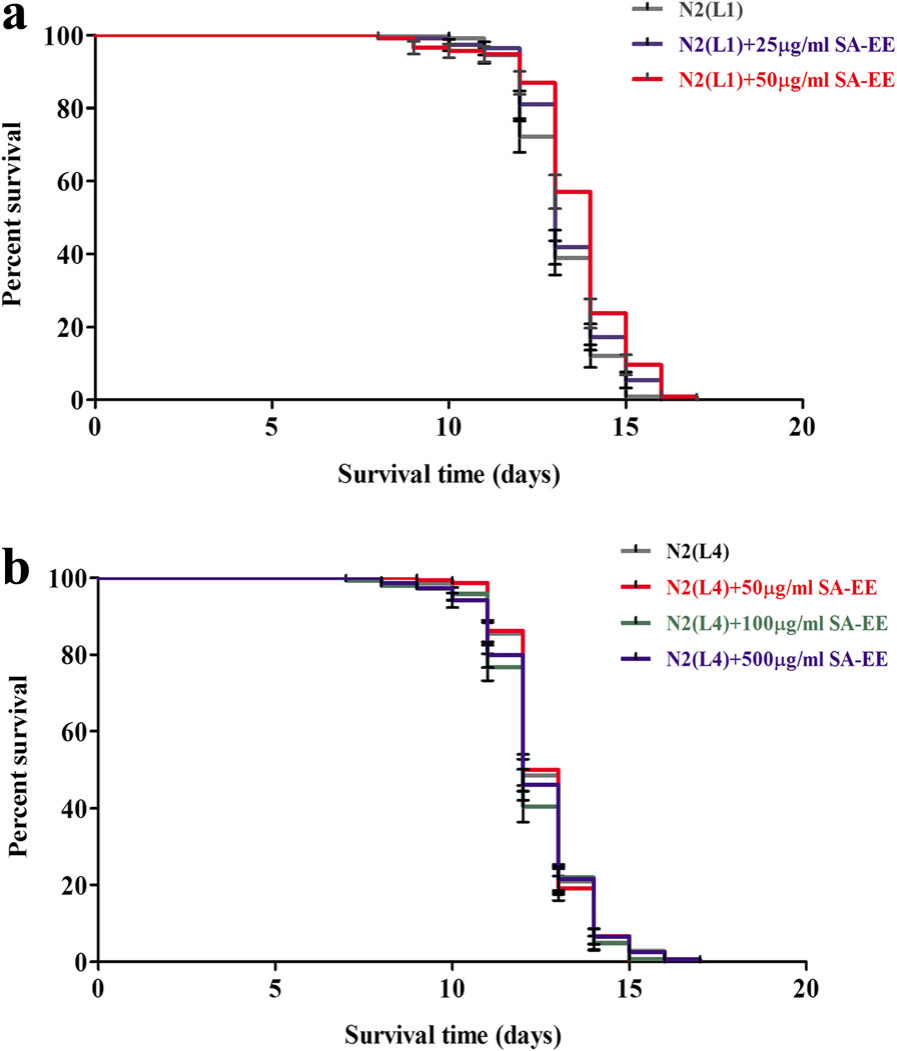
Effect of SA-EE on lifespan in *C. elegans.* Cumulative survival plots of wild-type N2 *C. elegans* at L1 (**a**) and L4 larval stages (**b**) grown at 25 °C treated with different concentrations of SA-EE or DMSO in control group. All data are shown as the mean ± SD from three independent experiments

To determine the anti-aging properties of SA-EE, we evaluated its effect on the lifespan of *C. elegans*. The results showed that SA-EE at a concentration of 50 μg/mL was capable of enhancing survival of wild-type N2 worms at L1 larval stage (Fig. 6a), but not the L4 larval stage (Fig. 6b), with the significant difference of survival rates between treated and control groups at *P* < 0.001. The mean lifespan of the SA-EE-treated L1-stage worms was 13.64 days that was slightly but significantly increased when compared to the control (Table 2).

**Table 2:** Results and statistical analyses of SA-EE treated *C. elegans* lifespan assay

Treatment	Number of worms	Mean lifespan (days)	Maximum lifespan (days)	Percentage of increased lifespan (vs control)	*P* value (vs control)
N2(L1) control	108	13.18 ± 0.11	16	–	–
N2(L1) + 25 μg/mL SA-EE	110	13.37 ± 0.12	16	1.44	0.232
N2(L1) + 50 μg/mL SA-EE	114	13.64 ± 0.14	17	3.49	0.010*
N2(L4) control	144	13.58 ± 0.11	18	–	–
N2(L4) + 50 μg/mL SA-EE	152	13.60 ± 0.09	16	0.15	0.873
N2(L4) + 100 μg/mL SA-EE	146	13.35 ± 0.12	17	−1.69	0.149
N2(L4) + 500 μg/mL SA-EE	154	13.37 ± 0.12	18	−1.55	0.516

Log-rank (Mantel-Cox) test was used in the analysis. *P < 0.05

### Phytochemical constituents of SA-EE

**Fig. 7:**
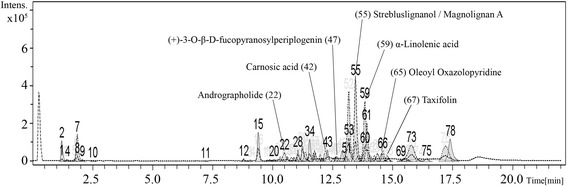
LC-MS chromatogram of SA-EE. The total ion chromatogram (TIC) of SA-EE was analyzed using LC-MS under positive electrospray ionization. Peak numbers indicate the proposed compounds as follows: andrographolide (22), carnosic acid (42), (+)-3-O-β-D-fucopyranosylperiplogenin (47), strebluslignanol or magnolignan A (55), α-linolenic acid (59), oleoyl oxazolopyridine (65), and taxifolin (67)

In the prediction of candidate compounds in SA-EE responsible for neuroprotective and/or anti-aging activities, we carried out LC-MS, and then chromatographic peaks were identified for candidate compounds based on the search of m/z values of molecular ion peaks in the positive mode [M + H]^+^ by comparison with databases and the literature. The LC-MS results revealed more than seventy isolated peaks in the chromatogram of SA-EE (Fig. 7), where the peaks of candidate compounds were annotated by number and detailed in Table 3. In this study, we reported four phytochemical compounds (peak no. 22, 42, 59, 65) proposed as active ingredients with neuroprotective and/or anti-aging properties along with three compounds (peak no. 47, 55, 67) that were previously reported in the literature.

**Table 3:** Proposed phytochemical constituents in SA-EE

Peak No.	Rt (min)	[M + H]^+^ (m/z)	Area (%)	Proposed compound	Theoretical mass	Mass error (ppm)	Database/ Ref
22	10.5	351.215	1.4	Andrographolide	350.209	4	METLIN
42	12.2	333.205	1.8	Carnosic acid	332.199	3	METLIN
47	12.7	537.307	0.6	(+)-3-O-β-D-fucopyranosylperiplogenin	536.298	3	[49]
55	13.5	301.141	9.8	Strebluslignanol, Magnolignan A	300.136	9	[50]
59	13.8	279.232	6.8	α-Linolenic acid	278.225	0	METLIN
65	14.5	385.292	1.1	Oleoyl Oxazolopyridine	384.277	19	METLIN
67	14.8	305.248	0.8	Taxifolin	304.250	30	[66]

## Discussion

Population aging is now affecting the entire world. The achievements of public health care programs, as well as the significant progress in socio-economic development over the past century has resulted in a higher proportion of people aged over 60 years than any other age group, which is progressing fastest in developing countries [19]. Besides labor shortages, the major unfavorable consequence of the growing aging population is the rising global burden of age-associated chronic non-communicable diseases, particularly neurodegenerative disorders [20, 21]. This leads to the need for long-term care from social services and imposes a great burden on health care costs, which has a long-term impact on socio-economic development. Effective management of these chronic diseases may serve as a way to cope with an aging society in the near future.

At present, there is no standard drug treatment for neurodegenerative diseases since these illnesses include a wide range of pathological conditions which are likely to involve different pathways of neuronal cell death, as well as several patterns of symptoms [22]. Current therapies only work for temporary symptomatic improvement and controlling the disease progression. Moreover, the debate over the clinical utility and cost-effectiveness of the major drugs used to treat AD, the most common neurodegenerative diseases (e.g. AChE inhibitors and N-methyl-D-aspartate (NMDA) receptor antagonists), has existed for decades as these drugs are not of benefit for all patients and long-term users are more likely to experience severe adverse drug reactions [1, 23, 24]. Nowadays the development of plant-based alternative medicine is challenging and promising in the search for new therapeutic targets and strategies for neurodegenerative diseases due to their good efficacy, and fewer side effects after long-term use [25].

A growing body of evidence suggests that glutamate-induced cell death in the brain may underlie the pathogenic mechanisms of chronic neurodegenerative disorders [2]. Glutamate is an endogenous excitatory neurotransmitter that plays a key role in a variety of normal brain functions. However at high extracellular concentration, it is neurotoxic and contributes to the development of certain neurodegenerative diseases such as AD, Parkinson’s disease (PD) and multiple sclerosis (MS). It was reported that glutamatergic neurotransmission was severely disrupted in the brains of individuals with AD [26], especially in the hippocampus and neocortex, where the activities of glutamine synthetase and glutamate transporter were found to be significantly decreased [27]. This would likely lead to increased extraneuronal glutamate concentrations and eventual cell death since both activities are required for glutamate clearance from the synapse. Moreover, prolonged exposure of high glutamate levels could in turn increase the production of toxic beta amyloid (A*β*), a well-known hallmark of AD, by regulating the amyloidogenic processing of the amyloid precursor protein (APP) [28]. Glutamate toxicity were also implicated in the degeneration of dopaminergic neurons in the substantia nigra, which is a hallmark of PD [2, 3], as well as in the pathophysiology of MS, a chronic inflammatory demyelinating disease of the CNS [29], and amyotrophic lateral sclerosis (ALS), a fatal disease caused by irreversible degeneration of motor neurons [30].

Glutamate-induced neurodegeneration can be mediated by two different pathways, receptor- and non-receptor-dependent. The classical receptor-initiated toxicity pathway, known as excitotoxicity, occurs through excessive stimulation of glutamate receptors especially the NMDA-type of receptor [31], whereas in the non-receptor-mediated oxidative toxicity pathway, high levels of glutamate reduce cystine uptake via the cystine/glutamate antiporter (system X_c_^−^), resulting in accumulation of ROS as a consequence of intracellular glutathione depletion, which contributes to increased lipid peroxidation, mitochondrial damage and ultimately cell death [32]. Nevertheless, the direct inhibition of NMDA receptors is likely to cause unacceptable clinical side effects, since the physiological activity of NMDA receptors is essential for normal neuronal function. Many NMDA receptor antagonists have disappointingly failed in clinical trials for a number of neurodegenerative disorders including AD [33–35], while memantine is the only drug among many which has been approved by the U.S. Food and Drug Administration (FDA) for the treatment of AD symptoms. However, the effect of this drug is still questionable, as it failed to show any statistically significant benefits in mild cases [36]. These studies suggest that the pathway of oxidative glutamate toxicity can be another promising therapeutic target for treatment of neurodegenerative diseases. A recent clinical trial supports this idea by showing that a well-known powerful antioxidant, vitamin E, has a beneficial effect in patients with mild to moderate AD, while memantine has no significant effect [37].

In this present study, we report, for the first time to our knowledge, the neuroprotective against glutamate toxicity and longevity effects of SA. The ethanolic extract of SA leaves could prevent glutamate-induced apoptotic cell death through the mechanisms underlying its antioxidant properties in the HT22 hippocampal cell line, and also extend the lifespan in *C. elegans*. Known as an important medicinal herb, SA has been used traditionally for various medicinal purposes, and reported to exhibit numerous biological properties, but its anti-aging effect as well as neuronal involvement has so far hardly been investigated. Previously, our group has reported that the plants possesses protective activities on glutamate toxic [38]. The recent finding of anti-PD effect in MPTP-treated C57BL/6 mice treated with SA extracts [10] prompted us to further explore the properties of this plant.

Among a number of cell models used in researching glutamate-mediated toxicity, the HT22 cell line can serve as an appropriate model system. As there is a lack of glutamate receptors in this cell line, the mechanism of cell death induced by glutamate is mainly due to oxidative stress from the reduction of glutathione levels, and a rise in intracellular ROS [39]. Excessive intracellular ROS levels have a detrimental effect that eventually contributes to cell death. Although the exact mechanism of oxidative-induced cell death in HT22 cell is not fully clear, increasing evidence suggests that the key step is translocation of the pro-apoptotic protein AIF from mitochondria into the nucleus, leading to triggering caspase-independent apoptosis [6, 32, 40, 41]. Accordingly, we confirmed in this study that release of AIF is required in the pathway of glutamate-induced oxidative damage. Our findings also showed that SA leaf extract could inhibit AIF nuclear translocation induced by glutamate, thereby preventing cells from undergoing apoptosis. Moreover, we observed that the majority of dead cells after glutamate treatment for 18 h in this study were positive for both annexin V and PI, indicating that they were likely cells in a late stage of apoptosis. This result is consistent to previous reports that cell death induced by glutamate was in time-dependent manner, with the glutamate treatment triggering apoptotic cell death relatively late ranging from 16 to 24 h post-treatment, although shorter periods of treatment can induce necrotic cell death [32].

SA has long been used as an ingredient in a popular Thai traditional formula for longevity [9]. However, so far no scientific evidence supports a longevity effect. Our present study showed that SA leaf extract could prolong survival and extend lifespan in *C. elegans* at the first larval stage (L1). In contrast, no effect on late larval stages (L4) was observed. This finding suggests that SA leaf extract exerts its longevity properties under normal conditions when the treatment occurs at an early age. Also this data supports the anti-aging benefit of SA-EE corresponding to its traditional usage. Even though there was only a slight increase in percentage of lifespan, this noticeable effect resulted from a single exposure, unlike some other studies in which the worms were freshly exposed to the extract approximately every day [42, 43].

Studies on the chemical constituents of SA have been shown that this plant contains a large number of cardiac glycosides [7]. Other compounds identified from SA include lignans, flavonoids, triterpenoids, and alkaloids [17, 44, 45]. However, previous phytochemical studies of isolated bioactive compounds from SA have focused on anti-hepatitis B [46–48], anti-cancer [49–51], and anti-microbial activities [52]. In the present study, we reported four candidate phytochemical compounds possibly responsible for neuroprotective and/or longevity effects of SA-EE. The proposed compounds include andrographolide, carnosic acid, α-linolenic acid, and oleoyl oxazolopyridine. Among these candidate compounds, carnosic acid is a molecule of interest as it has been shown to have antioxidative, anti-microbial, and anti-inflammatory properties [53, 54], in line with previous studies of beneficial effects exerted by SA. In addition, this compound protected neuronal cells from oxidative stress, A훽 and glutamate toxicities through activation of the Keap1/Nrf2 pathway [55–57]. Andrographolide has been reported to possess various pharmacological activities for treatment of cancer [58], inflammation [59], diabetes [60], and AD [61]. Moreover, derivative compounds bearing an oxazolopyridine core were identified as sirtuin activators and proposed to be used for treating a wide variety of diseases associated with aging [62]. Neuroprotective properties of α-linolenic acid, a plant-derived essential omega-3 polyunsaturated fatty acid, were also highlighted in several recent studies [63–65]. However, due to the complexity of crude extracts and limitation of LC-MS analysis, the compounds proposed here need to be confirmed in the near future with other identification techniques such as liquid chromatography-tandem mass spectrometry (LC-MS/MS) or quantitative HPLC. Nevertheless, at least three compounds from literature, which are (+)-3-O-β-D-fucopyranosylperiplogenin, strebluslignanol and/or magnolignan A, and taxifolin, were identified in this study, demonstrating an appreciable reliability of our LC-MS data.

## Conclusion

In conclusion, these findings demonstrate the neuroprotective action of SA leaf extracts in hippocampal neuronal cells which is mediated via inhibition of ROS accumulation, AIF nuclear translocation, and an increase of Nrf2 signaling. Significantly this extract extends *C. elegans* longevity. However, further studies of isolated bioactive components from SA leaf are required to elucidate the exact mechanisms involved in order to support the therapeutic potential of the plant extracts for age-related neurodegenerative disorders or as an anti-aging agent.

## Abbreviations

ABTS: 2,2′-Azino-bis(3-ethylbenzothiazoline-6-sulfonic acid) diammonium salt
AChE: Acetylcholinesterase
AD: Alzheimer’s disease
AIF: Apoptotic-inducing factor
CNS: Central nervous system
DPPH: 2,2-Diphenyl-1-picrylhydrazyl
EAAT3: Excitatory amino acid transporter 3
GCLM: Glutamate cysteine ligase complex modifier subunit
H_2_DCFDA: 2′, 7′-dichlorodihydrofluorescein diacetate
LDH: Lactate dehydrogenase
MTT: 3-(4,5-dimetylthiazol-2-yl)-2,5-diphenyltetrazoliumbromide
NMDA: N-methyl-D-aspartate
NQO1: NAD(P)H:quinone oxidoreductase 1
NRF2: Nuclear factor erythroid 2-related factor 2
PI: Propidium iodide
ROS: Reactive oxygen species
SA: *Streblus asper* Lour
SA-EE: *Streblus asper* leaf extracts
System Xc-: Cystine/Glutamate antiporter

## References

[1] Bond M, Rogers G, Peters J, Anderson R, Hoyle M, Miners A, Moxham T, Davis S, Thokala P, Wailoo A (2012). The effectiveness and cost-effectiveness of donepezil, galantamine, rivastigmine and memantine for the treatment of Alzheimer's disease (review of technology appraisal no. 111): a systematic review and economic model. Health Technol Assess.

[2] Sheldon AL, Robinson MB (2007). The role of glutamate transporters in neurodegenerative diseases and potential opportunities for intervention. Neurochem Int.

[3] Dong XX, Wang Y, Qin ZH (2009). Molecular mechanisms of excitotoxicity and their relevance to pathogenesis of neurodegenerative diseases. Acta Pharmacol Sin.

[4] Segovia G, Porras A, Del Arco A, Mora F (2001). Glutamatergic neurotransmission in aging: a critical perspective. Mech Ageing Dev.

[5] Chang L, Jiang CS, Ernst T (2009). Effects of age and sex on brain glutamate and other metabolites. Magn Reson Imaging.

[6] Tobaben S, Grohm J, Seiler A, Conrad M, Plesnila N, Culmsee C (2011). Bid-mediated mitochondrial damage is a key mechanism in glutamate-induced oxidative stress and AIF-dependent cell death in immortalized HT-22 hippocampal neurons. Cell Death Differ.

[7] Rastogi S, Kulshreshtha DK, Rawat AK (2006). Streblus Asper Lour. (Shakhotaka): a review of its chemical, pharmacological and Ethnomedicinal properties. Evid Based Complement Alternat Med.

[8] Singh SP (2015). A brief study on Strebulus asper L.-a review. Research journal of. Phytomedicine.

[9] Luanchoy S, Tiangkul S, Wongkrajang Y, Temsiririrkkul R, Peungvicha P, Nakornchai S (2014). Antioxidant activity of a Thai traditional formula for longevity. Mahidol. J Pharm Sci.

[10] Singsai K, Akaravichien T, Kukongviriyapan V, Sattayasai J. Protective effects of Streblus Asper leaf extract on H2O2-induced ROS in SK-N-SH cells and MPTP-induced Parkinson’s disease-like symptoms in C57BL/6 mouse. Evid Based Complement Alternat Med. 2015;2015. https://www.hindawi.com/journals/ecam/2015/970354/. 10.1155/2015/970354 PMC4698882 26798403

[11] Kakoti BB, Selvan VT, Saha P, Gupta M, Mazumder U. In Vivo and Invitro antioxidant properties of methanol extract of streblus asper lour. Journal of pharmaceutical and allied. Sciences. 2008;5(2).

[12] Ibrahim NM, Mat I, Lim V, Ahmad R (2013). Antioxidant activity and phenolic content of Streblus Asper leaves from various drying methods. Antioxidants.

[13] Chen H, Li J, Wu Q, Niu X-T, Tang M-T, Guan X-L, Li J, Yang R-Y, Deng S-P, X-J S (2012). Anti-HBV Activities of Streblus Asper and constituents of its roots. Fitoterapia.

[14] Faria A, Pestana D, Teixeira D, Azevedo J, De Freitas V, Mateus N, Calhau C (2010). Flavonoid transport across RBE4 cells: a blood-brain barrier model. Cell Mol Biol Lett.

[15] Yang Y, Bai L, Li X, Xiong J, Xu P, Guo C, Xue M (2014). Transport of active flavonoids, based on cytotoxicity and lipophilicity: an evaluation using the blood-brain barrier cell and Caco-2 cell models. Toxicol in Vitro.

[16] Lin JW, Chen JT, Hong CY, Lin YL, Wang KT, Yao CJ, Lai GM, Chen RM (2012). Honokiol traverses the blood-brain barrier and induces apoptosis of neuroblastoma cells via an intrinsic bax-mitochondrion-cytochrome c-caspase protease pathway. Neuro-Oncology.

[17] Verma V, Tripathi AC, Saraf SK (2016). Bioactive non-sterol triterpenoid from Streblus Asper: microwave-assisted extraction, HPTLC profiling, computational studies and neuro-pharmacological evaluation in BALB/c mice. Pharm Biol.

[18] Kim SM, Hwang IK, Yoo DY, Eum WS, Kim DW, Shin MJ, Ahn EH, Jo HS, Ryu EJ, Yong JI (2015). Tat-antioxidant 1 protects against stress-induced hippocampal HT-22 cells death and attenuate ischaemic insult in animal model. J Cell Mol Med.

[19] United Nations, Department of Economic and Social Affairs, Population Division. World Population Prospects: The 2017 Revision, Key Findings and Advance Tables 2017. https://esa.un.org/unpd/wpp/Publications/Files/WPP2017_KeyFindings.pdf. Accessed 21 Sept 2017.

[20] Brookmeyer R, Johnson E, Ziegler-Graham K, Arrighi HM (2007). Forecasting the global burden of Alzheimer's disease. Alzheimers Dement.

[21] Jacqmin-Gadda H, Alperovitch A, Montlahuc C, Commenges D, Leffondre K, Dufouil C, Elbaz A, Tzourio C, Menard J, Dartigues JF (2013). 20-year prevalence projections for dementia and impact of preventive policy about risk factors. Eur J Epidemiol.

[22] Gorman AM (2008). Neuronal cell death in neurodegenerative diseases: recurring themes around protein handling. J Cell Mol Med.

[23] Clegg A, Bryant J, Nicholson T, McIntyre L, De Broe S, Gerard K, Waugh N (2001). Clinical and cost-effectiveness of donepezil, rivastigmine and galantamine for Alzheimer's disease: a rapid and systematic review. Health Technol Assess.

[24] Loveman E, Green C, Kirby J, Takeda A, Picot J, Payne E, Clegg A (2006). The clinical and cost-effectiveness of donepezil, rivastigmine, galantamine and memantine for Alzheimer's disease. Health Technol Assess.

[25] Prasansuklab A, Tencomnao T (2013). Amyloidosis in Alzheimer's disease: the toxicity of amyloid Beta (a beta ), Mechanisms of Its Accumulation and Implications of Medicinal Plants for Therapy. Evid Based Complement Alternat Med.

[26] Masliah E, Alford M, DeTeresa R, Mallory M, Hansen L (1996). Deficient glutamate transport is associated with neurodegeneration in Alzheimer's disease. Ann Neurol.

[27] Butterfield DA, Pocernich CB (2003). The glutamatergic system and Alzheimer's disease: therapeutic implications. CNS Drugs.

[28] Revett TJ, Baker GB, Jhamandas J, Kar S (2013). Glutamate system, amyloid ss peptides and tau protein: functional interrelationships and relevance to Alzheimer disease pathology. J Psychiatry Neurosci.

[29] Kostic M, Zivkovic N, Stojanovic I (2013). Multiple sclerosis and glutamate excitotoxicity. Rev Neurosci.

[30] Rothstein JD, Van Kammen M, Levey AI, Martin LJ, Kuncl RW (1995). Selective loss of glial glutamate transporter GLT-1 in amyotrophic lateral sclerosis. Ann Neurol.

[31] Wang Y, Qin ZH (2010). Molecular and cellular mechanisms of excitotoxic neuronal death. Apoptosis.

[32] Fukui M, Song JH, Choi J, Choi HJ, Zhu BT (2009). Mechanism of glutamate-induced neurotoxicity in HT22 mouse hippocampal cells. Eur J Pharmacol.

[33] Lipton SA (2004). Failures and successes of NMDA receptor antagonists: molecular basis for the use of open-channel blockers like memantine in the treatment of acute and chronic neurologic insults. NeuroRx.

[34] Lipton SA (2004). Paradigm shift in NMDA receptor antagonist drug development: molecular mechanism of uncompetitive inhibition by memantine in the treatment of Alzheimer's disease and other neurologic disorders. J Alzheimers Dis.

[35] Jia Q, Deng Y, Qing H (2014). Potential therapeutic strategies for Alzheimer's disease targeting or beyond beta-amyloid: insights from clinical trials. Biomed Res Int.

[36] Alzheimer's Association. Fact sheet: Memantine (Namenda). 2006 https://www.alznyc.org/aboutalz/pdf/FSmemantine.pdf. Accessed 21 Sept 2017.

[37] Dysken MW, Sano M, Asthana S, Vertrees JE, Pallaki M, Llorente M, Love S, Schellenberg GD, McCarten JR, Malphurs J (2014). Effect of vitamin E and memantine on functional decline in Alzheimer disease: the TEAM-AD VA cooperative randomized trial. JAMA.

[38] Brimson JM, Brimson SJ, Brimson CA, Rakkhitawatthana V, Tencomnao T (2012). Rhinacanthus nasutus extracts prevent glutamate and amyloid-beta neurotoxicity in HT-22 mouse hippocampal cells: possible active compounds include Lupeol, Stigmasterol and beta-Sitosterol. Int J Mol Sci.

[39] Kritis AA, Stamoula EG, Paniskaki KA, Vavilis TD (2015). Researching glutamate - induced cytotoxicity in different cell lines: a comparative/collective analysis/study. Front Cell Neurosci.

[40] Zhang Y, Bhavnani BR (2006). Glutamate-induced apoptosis in neuronal cells is mediated via caspase-dependent and independent mechanisms involving calpain and caspase-3 proteases as well as apoptosis inducing factor (AIF) and this process is inhibited by equine estrogens. BMC Neurosci.

[41] Landshamer S, Hoehn M, Barth N, Duvezin-Caubet S, Schwake G, Tobaben S, Kazhdan I, Becattini B, Zahler S, Vollmar A (2008). Bid-induced release of AIF from mitochondria causes immediate neuronal cell death. Cell Death Differ.

[42] Bass TM, Weinkove D, Houthoofd K, Gems D, Partridge L (2007). Effects of resveratrol on lifespan in Drosophila Melanogaster and Caenorhabditis Elegans. Mech Ageing Dev.

[43] Honda Y, Tanaka M, Honda S (2010). Trehalose extends longevity in the nematode Caenorhabditis Elegans. Aging Cell.

[44] Li C, Huang C, Lu T, Wu L, Deng S, Yang R, Li J (2014). Tandem mass spectrometric fragmentation behavior of lignans, flavonoids and triterpenoids in Streblus Asper. Rapid Commun Mass Spectrom.

[45] Lu X, Li J, Huang C, Meng A, Zhu S (2009). Chemical constituents in leaves of Streblus Asper. Journal of Guangxi Normal University-Natural Science Edition.

[46] Li J, Huang Y, Guan XL, Li J, Deng SP, Wu Q, Zhang YJ, XJ S, Yang RY, Anti-hepatitis B (2012). Virus constituents from the stem bark of Streblus Asper. Phytochemistry.

[47] Li J, Meng AP, Guan XL, Li J, Wu Q, Deng SP, XJ S, Yang RY, Anti-hepatitis B (2013). Virus lignans from the root of Streblus Asper. Bioorg Med Chem Lett.

[48] Li LQ, Li J, Huang Y, Wu Q, Deng SP, XJ S, Yang RY, Huang JG, Chen ZZ, Li S (2012). Lignans from the heartwood of Streblus Asper and their inhibiting activities to hepatitis B virus. Fitoterapia.

[49] Ren Y, Chen W-L, Lantvit DD, Sass EJ, Shriwas P, Ninh TN, Chai H-B, Zhang X, Soejarto DD, Chen X (2016). Cardiac glycoside constituents of Streblus Asper with potential antineoplastic activity. J Nat Prod.

[50] Li J, Zhang YJ, Jin BF, XJ S, Tao YW, She ZG, Lin YC (2008). 1H and 13C NMR assignments for two lignans from the heartwood of Streblus Asper. Magn Reson Chem.

[51] Phutdhawong W, Donchai A, Korth J, Pyne SG, Picha P, Ngamkham J, Buddhasukh D (2004). The components and anticancer activity of the volatile oil from Streblus Asper. Flavour and fragrance journal.

[52] Nie H, Guan X-L, Li J, Zhang Y-J, He R-J, Huang Y, Liu B-M, Zhou D-X, Deng S-P, Chen H-C (2016). Antimicrobial lignans derived from the roots of Streblus Asper. Phytochem Lett.

[53] Birtic S, Dussort P, Pierre FX, Bily AC, Roller M (2015). Carnosic acid. Phytochemistry.

[54] Hou CW, Lin YT, Chen YL, Wang YH, Chou JL, Ping LY, Jeng KC (2012). Neuroprotective effects of carnosic acid on neuronal cells under ischemic and hypoxic stress. Nutr Neurosci.

[55] Satoh T, Kosaka K, Itoh K, Kobayashi A, Yamamoto M, Shimojo Y, Kitajima C, Cui J, Kamins J, Okamoto S (2008). Carnosic acid, a catechol-type electrophilic compound, protects neurons both in vitro and in vivo through activation of the Keap1/Nrf2 pathway via S-alkylation of targeted cysteines on Keap1. J Neurochem.

[56] Satoh T, Izumi M, Inukai Y, Tsutsumi Y, Nakayama N, Kosaka K, Shimojo Y, Kitajima C, Itoh K, Yokoi T (2008). Carnosic acid protects neuronal HT22 cells through activation of the antioxidant-responsive element in free carboxylic acid- and catechol hydroxyl moieties-dependent manners. Neurosci Lett.

[57] Rasoolijazi H, Azad N, Joghataei MT, Kerdari M, Nikbakht F, Soleimani M (2013). The protective role of carnosic acid against beta-amyloid toxicity in rats. ScientificWorldJournal.

[58] Rajagopal S, Kumar RA, Deevi DS, Satyanarayana C, Rajagopalan R (2003). Andrographolide, a potential cancer therapeutic agent isolated from Andrographis Paniculata. J Exp Ther Oncol.

[59] Li Y, He S, Tang J, Ding N, Chu X, Cheng L, Ding X, Liang T, Feng S, Rahman SU (2017). Andrographolide Inhibits Inflammatory Cytokines Secretion in LPS-Stimulated RAW264.7 Cells through Suppression of NF-kappaB/MAPK Signaling Pathway. Evid Based Complement Alternat Med.

[60] Zhang Z, Jiang J, Yu P, Zeng X, Larrick JW, Wang Y (2009). Hypoglycemic and beta cell protective effects of andrographolide analogue for diabetes treatment. J Transl Med.

[61] Serrano FG, Tapia-Rojas C, Carvajal FJ, Hancke J, Cerpa W, Inestrosa NC (2014). Andrographolide reduces cognitive impairment in young and mature AbetaPPswe/PS-1 mice. Mol Neurodegener.

[62] Villalba JM, de Cabo R, Alcain FJ (2012). A patent review of sirtuin activators: an update. Expert Opin Ther Pat.

[63] Blondeau N, Lipsky RH, Bourourou M, Duncan MW, Gorelick PB, Marini AM (2015). Alpha-linolenic acid: an omega-3 fatty acid with neuroprotective properties-ready for use in the stroke clinic?. Biomed Res Int.

[64] Shashikumar S, Pradeep H, Chinnu S, Rajini PS, Rajanikant GK (2015). Alpha-linolenic acid suppresses dopaminergic neurodegeneration induced by 6-OHDA in C. Elegans. Physiol Behav.

[65] Pan H, Piermartiri TC, Chen J, McDonough J, Oppel C, Driwech W, Winter K, McFarland E, Black K, Figueiredo T (2015). Repeated systemic administration of the nutraceutical alpha-linolenic acid exerts neuroprotective efficacy, an antidepressant effect and improves cognitive performance when given after soman exposure. Neurotoxicology.

[66] C L, X Z, Wang Z, X S, Xu Q (2010). Chemical constituents from the leaves of Streblus Asper [J]. Chinese traditional patent Medicine.

